# 2,3-Diamino­pyridinium 6-carb­oxy­pyridine-2-carboxyl­ate

**DOI:** 10.1107/S1600536811047647

**Published:** 2011-11-16

**Authors:** Mahsa Foroughian, Alireza Foroumadi, Behrouz Notash, Giuseppe Bruno, Hadi Amiri Rudbari, Hossein Aghabozorg

**Affiliations:** a Faculty of Chemistry, Islamic Azad University, North Tehran Branch, Tehran, Iran; b Drug Design & Development Research Center, Tehran University of Medical Sciences, Tehran, Iran; c Department of Chemistry, Shahid Beheshti University, G. C., Evin, Tehran 1983963113, Iran; d Dipartimento di Chimica Inorganica, Vill. S. Agata, Salita Sperone 31, Universita di Messina, 98166 Messina, Italy

## Abstract

The asymmetric unit of the title proton-transfer compound, C_5_H_8_N_3_^+^·C_7_H_4_NO_4_^−^, consists of one mono-deprotonated pyridine-2,6-dicarb­oxy­lic acid as anion and one protonated 2,3-diamino­pyridine as cation. The crystal packing shows extensive O—H⋯O, N—H⋯O and N—H⋯N hydrogen bonds. Thre are also several π–π inter­actions between the anions and also between the cations [centriod–centroid distances = 3.6634 (7), 3.7269 (7), 3.6705 (7) and 3.4164 (7) Å].

## Related literature

For background to proton-transfer compounds, see: Aghabozorg *et al.* (2008*b* ▶). For related structures, see: Aghabozorg *et al.* (2008*a* ▶, 2011*a* ▶,*b* ▶); Sharif *et al.* (2010 ▶).
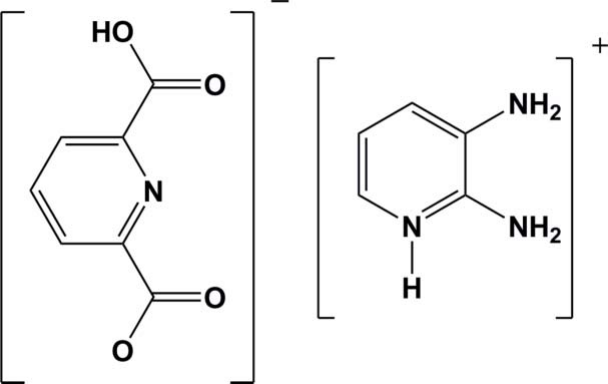


## Experimental

### 

#### Crystal data

C_5_H_8_N_3_^+^·C_7_H_4_NO_4_^−^
*M* *_r_* = 276.26
Triclinic, 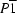

*a* = 6.9138 (1) Å
*b* = 8.3364 (2) Å
*c* = 11.2358 (2) Å
α = 81.448 (1)°
β = 73.831 (1)°
γ = 82.486 (1)°
*V* = 612.33 (2) Å^3^
*Z* = 2
Mo *K*α radiation
μ = 0.12 mm^−1^
*T* = 296 K
0.24 × 0.20 × 0.12 mm

#### Data collection

Bruker APEXII CCD diffractometer
Absorption correction: multi-scan (*SADABS*; Bruker, 2008 ▶) *T*_min_ = 0.706, *T*_max_ = 0.746
34839 measured reflections
2654 independent reflections
2348 reflections with *I* > 2σ(*I*)
*R*_int_ = 0.025

#### Refinement

*R*[*F* ^2^ > 2σ(*F* ^2^)] = 0.035
*wR*(*F* ^2^) = 0.107
*S* = 1.07
2654 reflections
194 parameters
H atoms treated by a mixture of independent and constrained refinement
Δρ_max_ = 0.30 e Å^−3^
Δρ_min_ = −0.17 e Å^−3^

### 

Data collection: *APEX2* (Bruker, 2008 ▶); cell refinement: *SAINT* (Bruker, 2008 ▶); data reduction: *SAINT*; program(s) used to solve structure: *SHELXS97* (Sheldrick, 2008 ▶); program(s) used to refine structure: *SHELXL97* (Sheldrick, 2008 ▶); molecular graphics: *SHELXTL* (Sheldrick, 2008 ▶); software used to prepare material for publication: *SHELXTL*.

## Supplementary Material

Crystal structure: contains datablock(s) I, global. DOI: 10.1107/S1600536811047647/bt5705sup1.cif

Structure factors: contains datablock(s) I. DOI: 10.1107/S1600536811047647/bt5705Isup2.hkl

Supplementary material file. DOI: 10.1107/S1600536811047647/bt5705Isup3.cml

Additional supplementary materials:  crystallographic information; 3D view; checkCIF report

## Figures and Tables

**Table 1:** Hydrogen-bond geometry (Å, °)

*D*—H⋯*A*	*D*—H	H⋯*A*	*D*⋯*A*	*D*—H⋯*A*
N4—H⋯O1^i^	0.86	1.94	2.7872 (12)	167
N2—H2*B*⋯O3^ii^	0.87 (2)	2.329 (19)	3.1108 (14)	150.0 (15)
N3—H3*A*⋯O2^i^	0.86	2.03	2.8674 (14)	163
N3—H3*B*⋯O2	0.86	2.17	2.9427 (14)	149
O4—H1⋯O1^iii^	0.89 (2)	1.75 (2)	2.5673 (12)	151.9 (18)
N2—H2*A*⋯O2	0.868 (19)	2.32 (2)	3.1290 (15)	156.0 (16)
N2—H2*A*⋯N1	0.868 (19)	2.491 (18)	3.0999 (14)	127.8 (15)

Symmetry codes: (i) 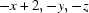; (ii) 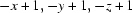; (iii) 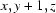.

**Fig. 1.**
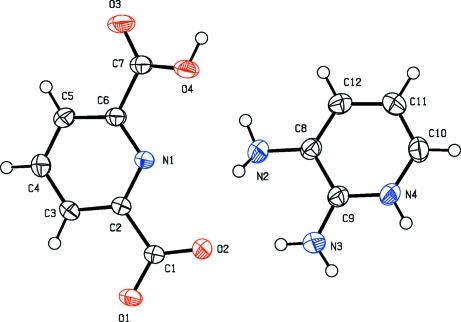
The molecular structure of the title compound. Displacement ellipsoids are drawn at 50% probability level.

**Fig. 2.**
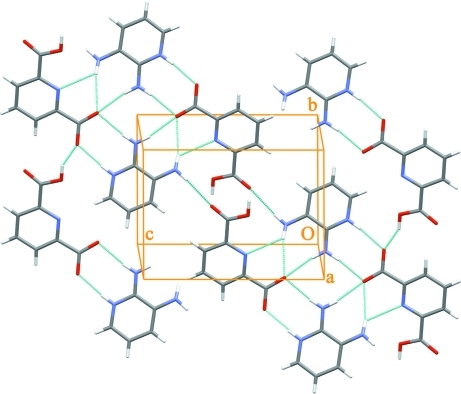
The packing diagram of the title compound showing hydrogen bonding interactions as blue dashed lines.

**Fig. 3.**
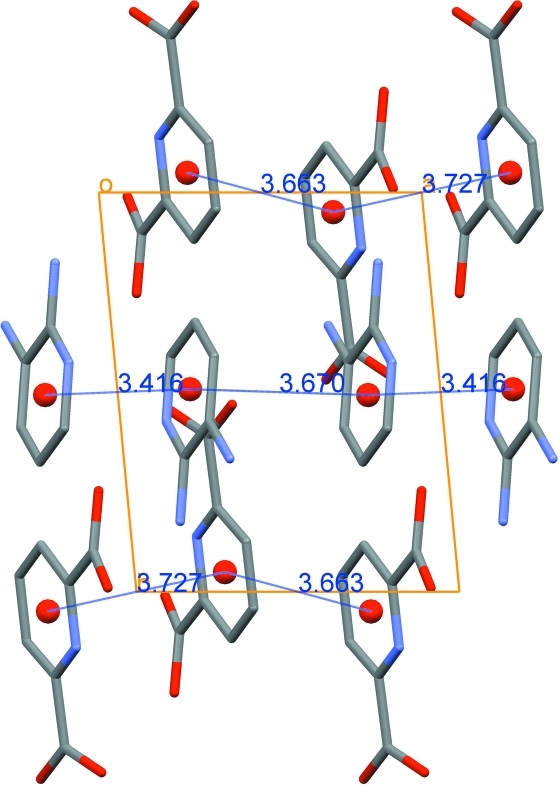
The packing diagram of the title compound showing π-π interactions between (py-2,6-dcH)- anions and between (dapyH)+ cations.

## supplementary crystallographic information

### Comment

Pyridinedicarboxylic acids are present in many natural products, such as vitamins, coenzymes, and alkaloids. Pyridine-2,6-dicarboxylic acid has been used to synthesize several proton transfer compounds in which pyridine-2,6-dicarboxylic acid acts as a monoacidic fragment (Aghabozorg *et al.* 2008*b*).In this regards, several organic bases were used such as propane-1,2-diamine (Aghabozorg *et al.* 2008*a*), *N^1^*,*N^1^*-dimethylpropane-1,2-diamine (Aghabozorg *et al.* 2011*a*), 2-amino-4-methylpyridinie (Sharif *et al.* 2010) and 2-amino-4-methylpyridine (Aghabozorg *et al.*, 2011*b*).

The structure of the title compounds contains one deprotonated pyridine-2,6-dicarboxylic acid as anion and one protonated 2,3-diaminopyridine as cation (Fig.1). The cations and anions are linked by several O—H···O, N—H···O and N—H···N hydrogen bonds with D···A distances ranging from 2.5673 (12) Å to 3.1290 (15) Å (Table.1 & Fig. 2). Furthermore, there are several π-π interactions which formed between (py-2,6-dcH)^-^ anions and also between (dapyH)^+^ cations with centriod-to-centroid distances = 3.6634 (7), 3.7269 (7), 3.6705 (7), 3.4164 (7) Å (Fig. 3). These hydrogen bonds and π-π interactions play important role in the stabilization of crystal packing.

### Experimental

The solution of pyridine-2,6-dicarboxylic acid (0.334 g, 2 mmol) in 7 ml water was added to solution of 2,3-diaminopyridine (0.218 g, 2 mmol) in 4 ml water in 1:1 molar ratios. The reaction mixture was stirred for 3hrs at room temprature. The colorless crystals of the title compound appeared after slow evaporation of solvent at room temperature.

### Refinement

The hydrogen atoms attached to O4, N2 were found in difference Fourier map and refined isotropically. The other H-atoms were included at calculated positions and treated as riding atoms: N—H = 0.86 Å for NH and NH_2_, C—H = 0.98 Å for aromatic CH hydrogen atoms. These H-atoms were refined with *Uiso*(H) = 1.2 × *Ueq*(parent atom).

### Crystal data

**Table:**

C_5_H_8_N_3_^+^·C_7_H_4_NO_4_^−^	*Z* = 2
*M**_r_* = 276.26	*F*(000) = 288
Triclinic, *P*1	*D*_x_ = 1.498 Mg m^−^^3^
Hall symbol: -P 1	Mo *K*α radiation, λ = 0.71073 Å
*a* = 6.9138 (1) Å	Cell parameters from 9842 reflections
*b* = 8.3364 (2) Å	θ = 2.9–30.0°
*c* = 11.2358 (2) Å	µ = 0.12 mm^−^^1^
α = 81.448 (1)°	*T* = 296 K
β = 73.831 (1)°	Irregular, colorless
γ = 82.486 (1)°	0.24 × 0.20 × 0.12 mm
*V* = 612.33 (2) Å^3^	

### Data collection

**Table:**

Bruker APEXII CCD diffractometer	2654 independent reflections
Radiation source: fine-focus sealed tube	2348 reflections with *I* > 2σ(*I*)
graphite	*R*_int_ = 0.025
φ and ω scans	θ_max_ = 27.0°, θ_min_ = 2.5°
Absorption correction: multi-scan (*SADABS*; Bruker, 2008)	*h* = −8→8
*T*_min_ = 0.706, *T*_max_ = 0.746	*k* = −10→10
34839 measured reflections	*l* = −14→14

### Refinement

**Table:**

Refinement on *F*^2^	Secondary atom site location: difference Fourier map
Least-squares matrix: full	Hydrogen site location: inferred from neighbouring sites
*R*[*F*^2^ > 2σ(*F*^2^)] = 0.035	H atoms treated by a mixture of independent and constrained refinement
*wR*(*F*^2^) = 0.107	*w* = 1/[σ^2^(*F*_o_^2^) + (0.060*P*)^2^ + 0.1194*P*] where *P* = (*F*_o_^2^ + 2*F*_c_^2^)/3
*S* = 1.07	(Δ/σ)_max_ < 0.001
2654 reflections	Δρ_max_ = 0.30 e Å^−^^3^
194 parameters	Δρ_min_ = −0.17 e Å^−^^3^
0 restraints	Extinction correction: *SHELXL97* (Sheldrick, 2008), Fc^*^=kFc[1+0.001xFc^2^λ^3^/sin(2θ)]^-1/4^
Primary atom site location: structure-invariant direct methods	Extinction coefficient: 0.00070 (17)

### Special details

**Table:**

Geometry. All s.u.'s (except the s.u. in the dihedral angle between two l.s. planes) are estimated using the full covariance matrix. The cell s.u.'s are taken into account individually in the estimation of s.u.'s in distances, angles and torsion angles; correlations between s.u.'s in cell parameters are only used when they are defined by crystal symmetry. An approximate (isotropic) treatment of cell s.u.'s is used for estimating s.u.'s involving l.s. planes.
Refinement. Refinement of *F*^2^ against ALL reflections. The weighted *R*-factor *wR* and goodness of fit *S* are based on *F*^2^, conventional *R*-factors *R* are based on *F*, with *F* set to zero for negative *F*^2^. The threshold expression of *F*^2^ > 2σ(*F*^2^) is used only for calculating *R*-factors(gt) *etc*. and is not relevant to the choice of reflections for refinement. *R*-factors based on *F*^2^ are statistically about twice as large as those based on *F*, and *R*- factors based on ALL data will be even larger.

### Fractional atomic coordinates and isotropic or equivalent isotropic displacement parameters (Å^2^)

**Table:**

	*x*	*y*	*z*	*U*_iso_*/*U*_eq_	
O3	0.64901 (16)	0.46889 (11)	0.60916 (9)	0.0508 (3)	
O2	0.91253 (17)	−0.01029 (10)	0.21751 (8)	0.0479 (3)	
O1	0.91229 (15)	−0.25266 (10)	0.32864 (8)	0.0419 (2)	
O4	0.83898 (16)	0.45238 (11)	0.41533 (8)	0.0480 (3)	
C8	0.72493 (16)	0.42375 (14)	0.08478 (10)	0.0310 (2)	
N4	0.85753 (15)	0.42597 (12)	−0.13478 (9)	0.0352 (2)	
H	0.9115	0.3742	−0.1986	0.042*	
N1	0.79718 (13)	0.13922 (10)	0.42873 (8)	0.0271 (2)	
C9	0.81096 (17)	0.33895 (14)	−0.02186 (10)	0.0314 (2)	
C10	0.8247 (2)	0.59072 (16)	−0.15453 (11)	0.0400 (3)	
H10	0.8605	0.6451	−0.2349	0.048*	
N3	0.84405 (18)	0.17690 (13)	−0.01308 (10)	0.0451 (3)	
H3A	0.8947	0.1294	−0.0793	0.054*	
H3B	0.8148	0.1199	0.0588	0.054*	
C11	0.7393 (2)	0.67446 (15)	−0.05574 (12)	0.0417 (3)	
H11	0.7130	0.7872	−0.0675	0.050*	
C12	0.69064 (19)	0.58993 (15)	0.06456 (11)	0.0379 (3)	
H12	0.6336	0.6481	0.1324	0.045*	
C7	0.73268 (17)	0.39063 (13)	0.52423 (10)	0.0319 (3)	
C6	0.72505 (16)	0.20977 (13)	0.53341 (10)	0.0276 (2)	
C2	0.79375 (15)	−0.02216 (12)	0.43816 (9)	0.0262 (2)	
C1	0.87808 (17)	−0.10003 (13)	0.31804 (10)	0.0299 (2)	
C3	0.71865 (17)	−0.11635 (13)	0.55079 (10)	0.0307 (2)	
H3	0.7201	−0.2286	0.5541	0.037*	
C4	0.64189 (18)	−0.04065 (14)	0.65777 (11)	0.0350 (3)	
H4	0.5889	−0.1007	0.7340	0.042*	
C5	0.64532 (18)	0.12568 (14)	0.64934 (10)	0.0332 (3)	
H5	0.5953	0.1802	0.7197	0.040*	
N2	0.67215 (18)	0.33369 (15)	0.20004 (10)	0.0402 (3)	
H2A	0.755 (3)	0.253 (2)	0.2184 (16)	0.057 (5)*	
H2B	0.625 (3)	0.397 (2)	0.2583 (17)	0.057 (5)*	
H1	0.840 (3)	0.560 (2)	0.4101 (17)	0.067 (5)*	

### Atomic displacement parameters (Å^2^)

**Table:**

	*U*^11^	*U*^22^	*U*^33^	*U*^12^	*U*^13^	*U*^23^
O3	0.0713 (7)	0.0303 (5)	0.0412 (5)	−0.0035 (4)	0.0066 (4)	−0.0163 (4)
O2	0.0818 (7)	0.0281 (4)	0.0250 (4)	0.0055 (4)	−0.0045 (4)	−0.0033 (3)
O1	0.0636 (6)	0.0200 (4)	0.0345 (5)	−0.0030 (4)	0.0009 (4)	−0.0068 (3)
O4	0.0736 (7)	0.0227 (4)	0.0374 (5)	−0.0120 (4)	0.0082 (4)	−0.0080 (3)
C8	0.0314 (5)	0.0338 (6)	0.0267 (5)	−0.0006 (4)	−0.0060 (4)	−0.0059 (4)
N4	0.0408 (5)	0.0369 (5)	0.0247 (5)	0.0027 (4)	−0.0051 (4)	−0.0064 (4)
N1	0.0332 (5)	0.0206 (4)	0.0257 (4)	−0.0017 (3)	−0.0049 (3)	−0.0040 (3)
C9	0.0327 (5)	0.0319 (6)	0.0285 (5)	0.0003 (4)	−0.0071 (4)	−0.0054 (4)
C10	0.0457 (7)	0.0385 (6)	0.0311 (6)	0.0024 (5)	−0.0092 (5)	0.0028 (5)
N3	0.0653 (7)	0.0318 (5)	0.0317 (5)	0.0031 (5)	−0.0038 (5)	−0.0069 (4)
C11	0.0500 (7)	0.0299 (6)	0.0420 (7)	0.0053 (5)	−0.0121 (5)	−0.0025 (5)
C12	0.0430 (6)	0.0356 (6)	0.0331 (6)	0.0044 (5)	−0.0069 (5)	−0.0110 (5)
C7	0.0378 (6)	0.0252 (5)	0.0312 (5)	−0.0023 (4)	−0.0046 (4)	−0.0076 (4)
C6	0.0303 (5)	0.0233 (5)	0.0283 (5)	−0.0015 (4)	−0.0050 (4)	−0.0058 (4)
C2	0.0303 (5)	0.0211 (5)	0.0267 (5)	−0.0005 (4)	−0.0072 (4)	−0.0035 (4)
C1	0.0384 (6)	0.0220 (5)	0.0280 (5)	−0.0016 (4)	−0.0064 (4)	−0.0046 (4)
C3	0.0384 (6)	0.0209 (5)	0.0309 (5)	−0.0033 (4)	−0.0073 (4)	−0.0008 (4)
C4	0.0421 (6)	0.0314 (6)	0.0269 (5)	−0.0057 (5)	−0.0030 (4)	0.0011 (4)
C5	0.0395 (6)	0.0313 (6)	0.0257 (5)	−0.0022 (4)	−0.0017 (4)	−0.0077 (4)
N2	0.0489 (6)	0.0394 (6)	0.0263 (5)	0.0006 (5)	−0.0022 (4)	−0.0043 (4)

### Geometric parameters (Å, °)

**Table:**

O3—C7	1.2041 (14)	N3—H3A	0.8600
O2—C1	1.2401 (14)	N3—H3B	0.8600
O1—C1	1.2574 (13)	C11—C12	1.4008 (18)
O4—C7	1.3088 (14)	C11—H11	0.9300
O4—H1	0.89 (2)	C12—H12	0.9300
C8—C12	1.3702 (17)	C7—C6	1.5028 (15)
C8—N2	1.3760 (15)	C6—C5	1.3862 (15)
C8—C9	1.4262 (15)	C2—C3	1.3903 (14)
N4—C9	1.3414 (14)	C2—C1	1.5185 (14)
N4—C10	1.3575 (16)	C3—C4	1.3811 (16)
N4—H	0.8600	C3—H3	0.9300
N1—C6	1.3343 (14)	C4—C5	1.3786 (16)
N1—C2	1.3365 (13)	C4—H4	0.9300
C9—N3	1.3338 (15)	C5—H5	0.9300
C10—C11	1.3512 (18)	N2—H2A	0.868 (19)
C10—H10	0.9300	N2—H2B	0.87 (2)
			
C7—O4—H1	112.4 (12)	O3—C7—C6	122.78 (10)
C12—C8—N2	124.24 (10)	O4—C7—C6	112.99 (9)
C12—C8—C9	117.42 (10)	N1—C6—C5	123.76 (10)
N2—C8—C9	118.25 (10)	N1—C6—C7	117.67 (9)
C9—N4—C10	124.00 (10)	C5—C6—C7	118.57 (10)
C9—N4—H	118.0	N1—C2—C3	122.83 (9)
C10—N4—H	118.0	N1—C2—C1	116.38 (9)
C6—N1—C2	117.28 (9)	C3—C2—C1	120.79 (9)
N3—C9—N4	119.21 (10)	O2—C1—O1	124.63 (10)
N3—C9—C8	122.31 (10)	O2—C1—C2	118.57 (9)
N4—C9—C8	118.47 (10)	O1—C1—C2	116.78 (9)
C11—C10—N4	119.02 (11)	C4—C3—C2	118.99 (10)
C11—C10—H10	120.5	C4—C3—H3	120.5
N4—C10—H10	120.5	C2—C3—H3	120.5
C9—N3—H3A	120.0	C5—C4—C3	118.73 (10)
C9—N3—H3B	120.0	C5—C4—H4	120.6
H3A—N3—H3B	120.0	C3—C4—H4	120.6
C10—C11—C12	119.37 (11)	C4—C5—C6	118.40 (10)
C10—C11—H11	120.3	C4—C5—H5	120.8
C12—C11—H11	120.3	C6—C5—H5	120.8
C8—C12—C11	121.69 (11)	C8—N2—H2A	118.7 (12)
C8—C12—H12	119.2	C8—N2—H2B	110.6 (12)
C11—C12—H12	119.2	H2A—N2—H2B	113.8 (16)
O3—C7—O4	124.23 (10)		
			
C10—N4—C9—N3	178.04 (12)	O3—C7—C6—C5	−10.12 (18)
C10—N4—C9—C8	−1.15 (17)	O4—C7—C6—C5	169.81 (11)
C12—C8—C9—N3	−177.65 (11)	C6—N1—C2—C3	0.21 (16)
N2—C8—C9—N3	−0.97 (17)	C6—N1—C2—C1	−179.54 (9)
C12—C8—C9—N4	1.52 (16)	N1—C2—C1—O2	−11.91 (15)
N2—C8—C9—N4	178.20 (10)	C3—C2—C1—O2	168.34 (11)
C9—N4—C10—C11	−0.31 (19)	N1—C2—C1—O1	166.52 (10)
N4—C10—C11—C12	1.3 (2)	C3—C2—C1—O1	−13.22 (16)
N2—C8—C12—C11	−176.99 (12)	N1—C2—C3—C4	0.76 (17)
C9—C8—C12—C11	−0.54 (18)	C1—C2—C3—C4	−179.51 (10)
C10—C11—C12—C8	−0.9 (2)	C2—C3—C4—C5	−0.98 (17)
C2—N1—C6—C5	−0.96 (16)	C3—C4—C5—C6	0.29 (18)
C2—N1—C6—C7	178.62 (9)	N1—C6—C5—C4	0.71 (18)
O3—C7—C6—N1	170.28 (12)	C7—C6—C5—C4	−178.86 (10)
O4—C7—C6—N1	−9.79 (15)		

### Hydrogen-bond geometry (Å, °)

**Table:**

*D*—H···*A*	*D*—H	H···*A*	*D*···*A*	*D*—H···*A*
N4—H···O1^i^	0.86	1.94	2.7872 (12)	167.
N2—H2B···O3^ii^	0.87 (2)	2.329 (19)	3.1108 (14)	150.0 (15)
N3—H3A···O2^i^	0.86	2.03	2.8674 (14)	163.
N3—H3B···O2	0.86	2.17	2.9427 (14)	149.
O4—H1···O1^iii^	0.89 (2)	1.75 (2)	2.5673 (12)	151.9 (18)
N2—H2A···O2	0.868 (19)	2.32 (2)	3.1290 (15)	156.0 (16)
N2—H2A···N1	0.868 (19)	2.491 (18)	3.0999 (14)	127.8 (15)

Symmetry codes: (i) −*x*+2, −*y*, −*z*; (ii) −*x*+1, −*y*+1, −*z*+1; (iii) *x*, *y*+1, *z*.
